# Single-cell transcriptome profiling of m^6^A regulator-mediated methylation modification patterns in elderly acute myeloid leukemia patients

**DOI:** 10.1186/s43556-024-00234-7

**Published:** 2024-12-06

**Authors:** Zhe Wang, Xin Du, Peidong Zhang, Meiling Zhao, Tianbo Zhang, Jiang Liu, Xiaolan Wang, Doudou Chang, Xiaxia Liu, Sicheng Bian, Xialin Zhang, Ruijuan Zhang

**Affiliations:** 1 Department of Gynecology, First Hospital of Shanxi Medical University, Taiyuan, Shanxi 030001 China; 2 Department of Hematology, Shanxi Bethune Hospital, Third Hospital of Shanxi Medical University, Shanxi Academy of Medical Sciences, Tongji Shanxi Hospital, Taiyuan, 030032 China; 3 State Key Laboratory of Biotherapy and Cancer Center, West China Hospital, Sichuan University, Chengdu, 610000 China; 4 Department of Hematology, Linfen Central Hospital, Linfen, 041000 China; 5 Department of Medicine, Case Western Reserve University, Cleveland, OH 44106 USA

**Keywords:** M^6^A, Acute myeloid leukemia, Malignant differentiation, WNT signaling, Single-cell RNA-seq

## Abstract

Millions of people worldwide die of acute myeloid leukaemia (AML) each year. Although N6-methyladenosine (m^6^A) modification has been reported to regulate the pathogenicity of AML, the mechanisms by which m^6^A induces dysfunctional hematopoietic differentiation in elderly AML patients remain elusive. This study elucidates the mechanisms of the m^6^A landscape and the specific roles of m^6^A regulators in hematopoietic cells of elderly AML patients. Notably, fat mass and obesity-associated protein (*FTO*) was found to be upregulated in hematopoietic stem cells (HSCs), myeloid cells, and T-cells, where it inhibits their differentiation via the WNT signaling pathway. Additionally, elevated YT521-B homology domain family proteins 2 (*YTHDF2*) expression in erythrocytes was observed to negatively regulate differentiation through oxidative phosphorylation, resulting in leukocyte activation. Moreover, *IGF2BP2* was significantly upregulated in myeloid cells, contributing to an aberrant chromosomal region and disrupted oxidative phosphorylation. m^6^A regulators were shown to induce abnormal cell-cell communication within hematopoietic cells, mediating ligand-receptor interactions across various cell types through the HMGB1-mediated pathway, thereby promoting AML progression. External validation was conducted using an independent single-cell RNA sequencing (scRNA-Seq) dataset. The THP-1 and MV411 cell lines were utilized to corroborate the m^6^A regulator profile; in vitro experiments involving short hairpin RNA (shRNA) targeting *FTO* demonstrated inhibition of cell proliferation, migration, and oxidative phosphorylation, alongside induction of cell cycle arrest and apoptosis. In summary, these findings suggest that the upregulation of m^6^A regulators in HSCs, erythrocytes, myeloid cells, and T-cells may contribute to the malignant differentiation observed in AML patients. This research provides novel insights into the pathogenesis of AML in elderly patients and identifies potential therapeutic targets.

**Supplementary Information:**

The online version contains supplementary material available at 10.1186/s43556-024-00234-7.

## Introduction

Acute myeloid leukaemia (AML) is a haematological malignancy characterized by strong heterogeneity and high mortality, and the incidence rate of AML continues to increase year by year [1]. Approximately 75% of patients affected by this disease are aged 65 and older [2]. Currently, the composite complete remission (cCR) rate in older adults with AML is only 17–66%, and the 5-year overall survival (OS) remains poor, reflecting the adverse disease biology and clinical symptoms of this population [3–5].

Numerous studies have identified that RNA modification is a posttranscriptional change in the chemical composition of RNA molecules, has the potential to alter diverse biological functions [6]. N6-methyladenosine (m^6^A) is the most prevalent modification found in eukaryotic mRNA; m^6^A can be stalled by methyltransferases (writers), namely, *METTL3*, *METTL14* and *WTAP*, reversed by demethylases (erasers), including *FTO* and *ALKBH5*, and recognized by m^6^A binding proteins (readers), such as *YTHDC1/2*, *YTHDF1-3* and *IGF2BP1-3* [7].

Recently, it has been reported that the dysregulation of m^6^A modification is involved in the occurrence and development of various cancers [8]. For instance, *METTL3* promotes the translation of the downstream genes *MYC*,* BCL2*, and *PTEN* through m^6^A modification and inhibits the *pAKT* pathway, while *METTL14* promotes the expression of the target genes *MYB* and *MYC*, thus promoting cell proliferation and inhibiting differentiation/apoptosis [9, 10]. Additionally, *FTO* can enhance the stability of *MYC* and *CEBPA* mRNA, thereby promoting cell proliferation [11], and *METTL14* and *YTHDF2* promote the self-renewal of leukaemia stem cells (LSCs)/leukaemia initiating cells (LICs), while *ALKBH5K* promotes LSC proliferation and induces apoptosis [10, 12, 13]. Moreover, m^6^A regulators are closely associated with the survival of AML patients [14].

The cellular hierarchy in AML patients is probably similar to the normal haematopoietic differentiation hierarchy, accompanied by functional impairment of various cell types [15]. The balance between the proliferation and differentiation of haematopoietic stem cells (HSCs), Myeloids, TCells and BCells is dysfunctional in AML patients [16, 17]. Furthermore, the immune function of Myeloids, TCells and BCells is changed in AML patients, especially the transformation of erythrocytes into immunosuppressive myeloid cells [17, 18]. The signaling pathways and metabolism of each cell type are also altered [19, 20]. At present, the regulatory mechanisms underlying the aberrant differentiation of AML by m^6^A regulators remain unclear. Single-cell RNA sequencing (scRNA-seq) can potentially be used to solve this problem by identifying the unique characteristics of each cell type in tissue samples and providing a more detailed understanding of intercellular communication networks [21].

In this study, we revealed a cell-type-specific m^6^A modification pattern in elderly patients with AML involving the distinct roles of *FTO* in different cell types, including HSCs, Myeloids and TCells. Importantly, we observed that the elevated expression of *YTHDF2* in Erythrocytes is involved in regulating erythrocyte differentiation. Additionally, CellChat analysis confirmed alterations in ligand-receptor pairs. The objective of this study is to identify novel therapeutic targets and to facilitate the development of more comprehensive treatment strategies for AML patients. Our study also aims to provide new approaches for enhancing clinical responses to immunotherapy, characterizing the tumor immune phenotypes of diverse cell types, and advancing personalized immunotherapy for AML.

## Results

### Distribution and reduction profiles of m6A regulator pattern through single-cell transcriptome atlas in AML patients

**Fig. 1:**
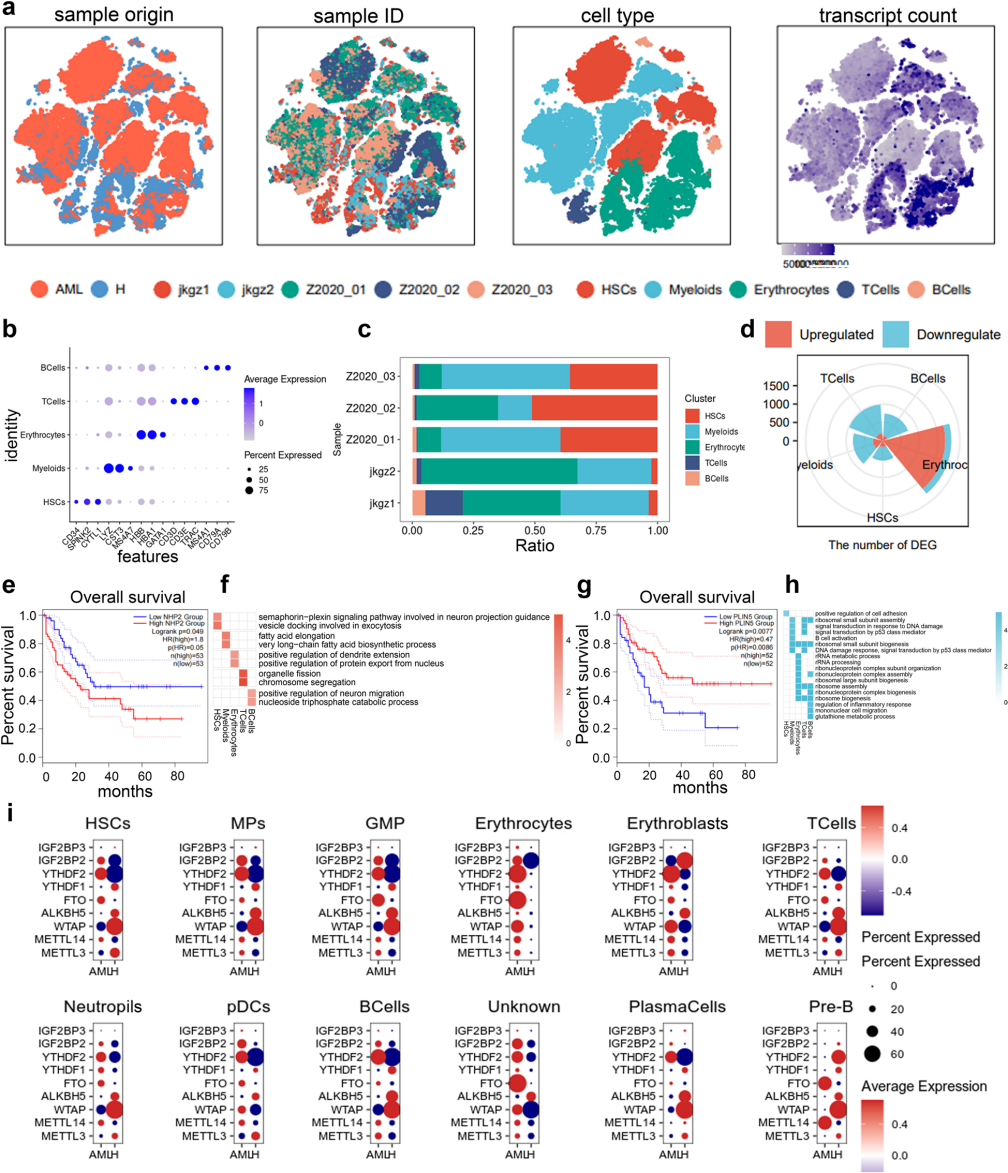
Diverse cell types in the AML delineated by Single-cell RNA-seq(scRNA-seq) analysis. **a** t-Distributed Stochastic Neighbor Embedding(tSNE) of the 54,809 cells profiled here, with each cell color coded for (left to right): its sample type of origin (malignant or non-malignant), the corresponding patient, the associated cell type and the unique molecular identifier (UMI) detected in that cell (log scale as defined in the inset). **b** The plot shows identified cell types and annotated using classical marker genes. **c** Data of the five cell clusters of 54,809 cells from 5 samples (from left to right): the fraction of cells originating from each patient. **d** Differential expression of genes in different cell types of AML patients compared with control samples. **e** and **g** Kaplan - Meier curves showing progression-free survival in GEPIA 2 in AML Cohort stratified according to high vs. low expression of *NHP2* (**e**) and *PLIN5* (**g**). **f** and **h** Heatmap showing the representative gene ontology enriched in upregulated (**f**) or downregulated (**h**) genes in each cell type (*p* < 0.05). **i** Differential expression of N6-methyladenosine (m^6^A) regulators in different cell types of AML patients compared with control samples from respective cell-type assignments; The size of the dots indicates the average multiple of difference, and the color of the dots indicates up-regulated (red) or down-regulated (purple)

To understand the cellular diversity and molecular features in AML patients, we used standard bone marrow (BM) aspiration methods to isolate hematocytes from the BM of three AML patients and two control individuals. We analyzed scRNA-seq datasets consisting of 54,809 cells, of which 42,953 were derived from AML samples. To visualize the cluster analysis, we performed uniform manifold approximation and projection (UMAP) and t-distributed stochastic neighbor embedding (t-SNE) using the RunTSNE function in Seurat. The visualizations included the sample type of origin (malignant or non-malignant), the corresponding patient, the associated cell type, and the unique molecular identifier (UMI) detected in each cell (Fig. 1a). Five known cell types were identified based on previously reported cell markers and other AML single-cell sequencing results [22], including 18,491 HSCs corresponding to cell markers *CD34*, *SPINK2*, and *CYTL1*, 20,274 Myeloids corresponding to cell markers *LYZ*, *CST3*, and *MS4A7*, 13,640 erythrocytes corresponding to cell markers *HBB*, *HBA1*, and *GATA1*, 1459 TCells corresponding to cell markers *CD3D*, *CD3E*, and *TRAC*, and 945 B cells corresponding to cell markers *MS4A1*, *CD79A*, and *CD79B* (Fig. 1b) (Fig. S1). Analysis of cell proportions indicated that HSCs, erythroblasts, macrophages (Mps), and B cells were more abundant in the AML patient group (Fig. 1c).

To further explore how diverse cell types impact AML progression, we examined transcriptional alterations in AML-derived cell types. We found that differentially expressed genes (DEGs) in erythrocytes were predominantly upregulated in AML samples compared to healthy samples, while others were downregulated (Fig. 1d). In AML compared to normal group, the top dysregulated genes across the five diverse cell types were identified (Fig. S1). We also identified DEGs in AML samples associated with patient prognosis. High-risk scores based on *NHP2* expression correlated with lower survival rates (Fig. 1e), while higher *PLIN5* expression correlated with higher survival rates (Fig. 1g). Notably, functional enrichment analysis revealed that hematopoietic stem cells (HSCs) in AML samples were associated with chemical synaptic transmission and mitochondrial homeostasis. Erythrocytes were linked to the regulation of dendrite extension and the positive regulation of protein export from the nucleus. T cells were associated with chromosome segregation, while B cells were linked to the apoptotic signaling pathway (Fig. 1f and h).

To understand the cellular features and mechanisms of m^6^A regulators in the bone marrow (BM) of AML patients, we annotated using classical marker genes. The marker genes for the cell populations are clearly shown. We then analyzed the expression levels of different m^6^A regulators (Fig. S3a) and defined 23 regulator signatures using the AddModuleScore. The expression of m^6^A regulators across diverse cell types was illustrated using a UMAP plot based on gene expression profiles. Additionally, we measured the percentage of m^6^A expression in each cell type in AML (Fig. S2).

To elucidate the mechanism of m^6^A regulation in diverse cell types in AML patient samples, we identified DEGs of m^6^A regulators. We found that *FTO*, *YTHDF2*, and *IGF2BP2* were mainly upregulated, while *WTAP*, *ALKBH5*, and *YTHDF1* were mainly downregulated in diverse cell types of AML (Fig. 1i). These findings suggest the intricate role of dysregulated m^6^A regulator function in AML progression.

### m6A regulators are involved in malignant differentiation in diverse cell types in AML patients

**Fig. 2:**
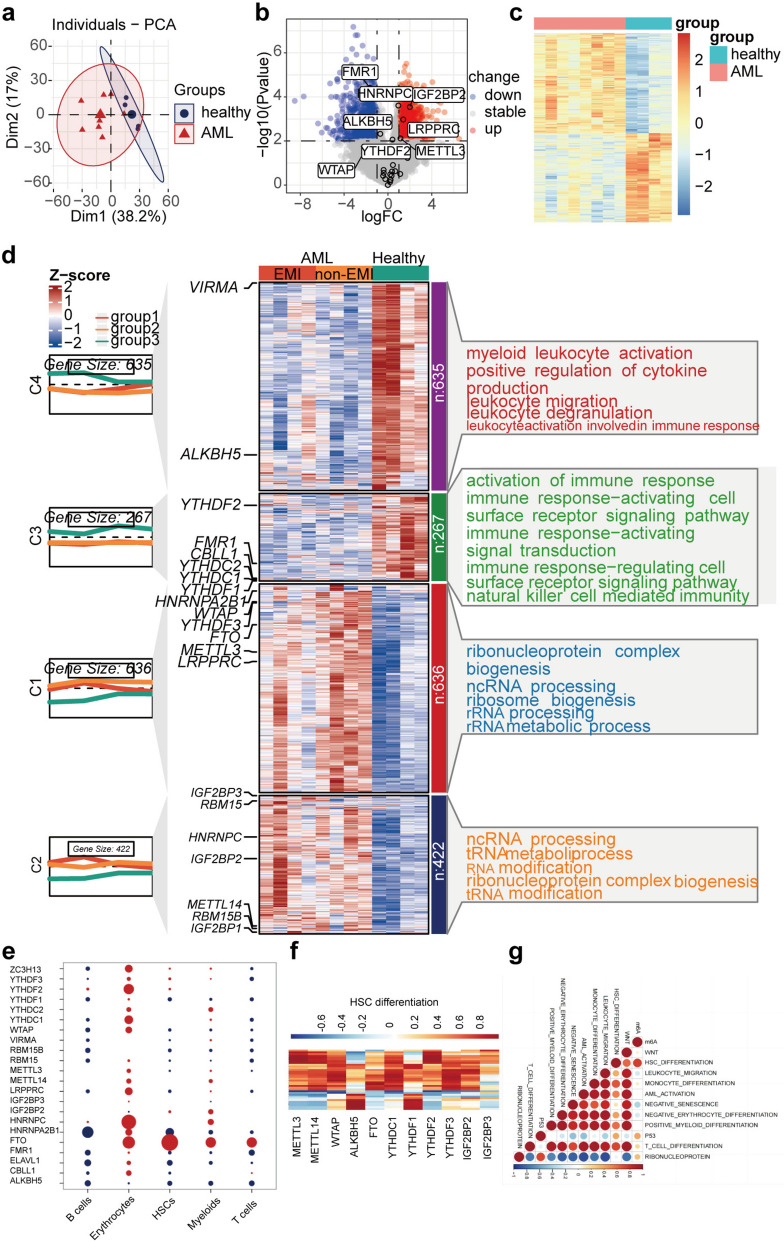
Expression patterns of m^6^A regulator in AML patients and healthy individuals. **a** Principal Component Analysis (PCA) was employed on 23 m6A regulators to differentiate between tumor and normal samples. **b** Volcano-plot representation of differentially expressed genes (DEGs). Red, up-regulation; blue, down-regulation. **c** Heatmap of the DEGs between the gene clusters and different clinical data was shown in the annotation. **d** Expression patterns of m^6^A regulators in AML patients and healthy individuals. Heatmap of DEGs between AML and control groups; the m^6^A regulators in each module were annotated; the line graph showed the trend in the gene module expression, the text on the right showed the enriched pathways for each module gene. **e** The DEGs patterns of m^6^A regulators of diverse cell types. **f** Gene set variation analysis (GSVA) enrichment analysis showing the association between m^6^A regulators and HSC differentiation in AML. **g** Spearman’s correlation was used to analyze the correlation between the m^6^A regulators and classical AML related pathways. Red, positive correlation; blue, negative correlation

To define gene expression changes globally and at the cellular level, we performed bulk RNA-seq on AML and control samples. Unsupervised clustering revealed two distinct patterns (Fig. 2a). In AML samples, *HNANPC*, *IGF2BP2*, *METTL3*, and *LRPPRC* were upregulated, while *FMR1* was downregulated (Fig. 2b).

To explore these m^6^A modification phenotypes, we focused on control and AML groups. Unsupervised clustering showed distinct patterns of m^6^A regulator expression (Fig. 2c). Further analysis across four AML subtypes (C1-C4) revealed that C2 contained *IGF2BP3*, *RBM15*, *HNRNPC*, *IGF2BP2*, *METTL14*, *RBM15B*, and *IGF2BP1*; C3 contained *YTHDF2*, *FMR1*, *CBLL1*, *YTHDC2*, *YTHDC1*, and *YTHDF1*; C1 contained *HNRNPA2B1*, *WTAP*, *YTHDF3*, *FTO*, *METTL3*, and *LRPPRC*; and C4 contained *VIRMA* and *ALKBH5*.

Based on mRNA profiles, we examined differences in m^6^A regulators between clusters C2 and C3, focusing on pathways related to chromosomal abnormalities and cellular immune function. We found that most pathways related to immune response activation, such as those involving immune response-activating cells and signal transduction, were downregulated in C3. This suggests that the dysfunction in immune pathways in C3 may be due to m^6^A regulators like *YTHDF2* (Fig. 2d).

Next, to reveal the differential expression of m^6^A molecules between the AML and control groups, we found that *FTO* was overexpressed in all cell types, *YTHDF2* in erythrocytes, and *IGF2BP2* in myeloid cells (Fig. 2e). To study the link between m^6^A regulation and AML progression, we performed GSEA-based functional enrichment analysis and correlated m^6^A regulators with AML-related pathways. Our analysis showed that upregulated m^6^A regulator genes were associated with HSC differentiation, the p53 pathway, ribonucleoprotein complexes, T-cell differentiation, monocyte differentiation, AML activation, and the Wnt pathway (*r* > 0.2). Conversely, downregulated m^6^A regulator genes were associated with negative senescence, negative erythrocyte differentiation, and leukocyte migration (*r* < −0.2) (Fig. 2g). Additionally, our findings were supported by further analyses presented (Fig. S3c and Fig. S3d), which detail the correlations between m^6^A regulators and specific AML pathways (Fig. 2f).

### FTO cell-type-specific upregulation impaired haemocyte differentiation and suppressed oxidative phosphorylation metabolism via WNT signaling in HSCs

**Fig. 3:**
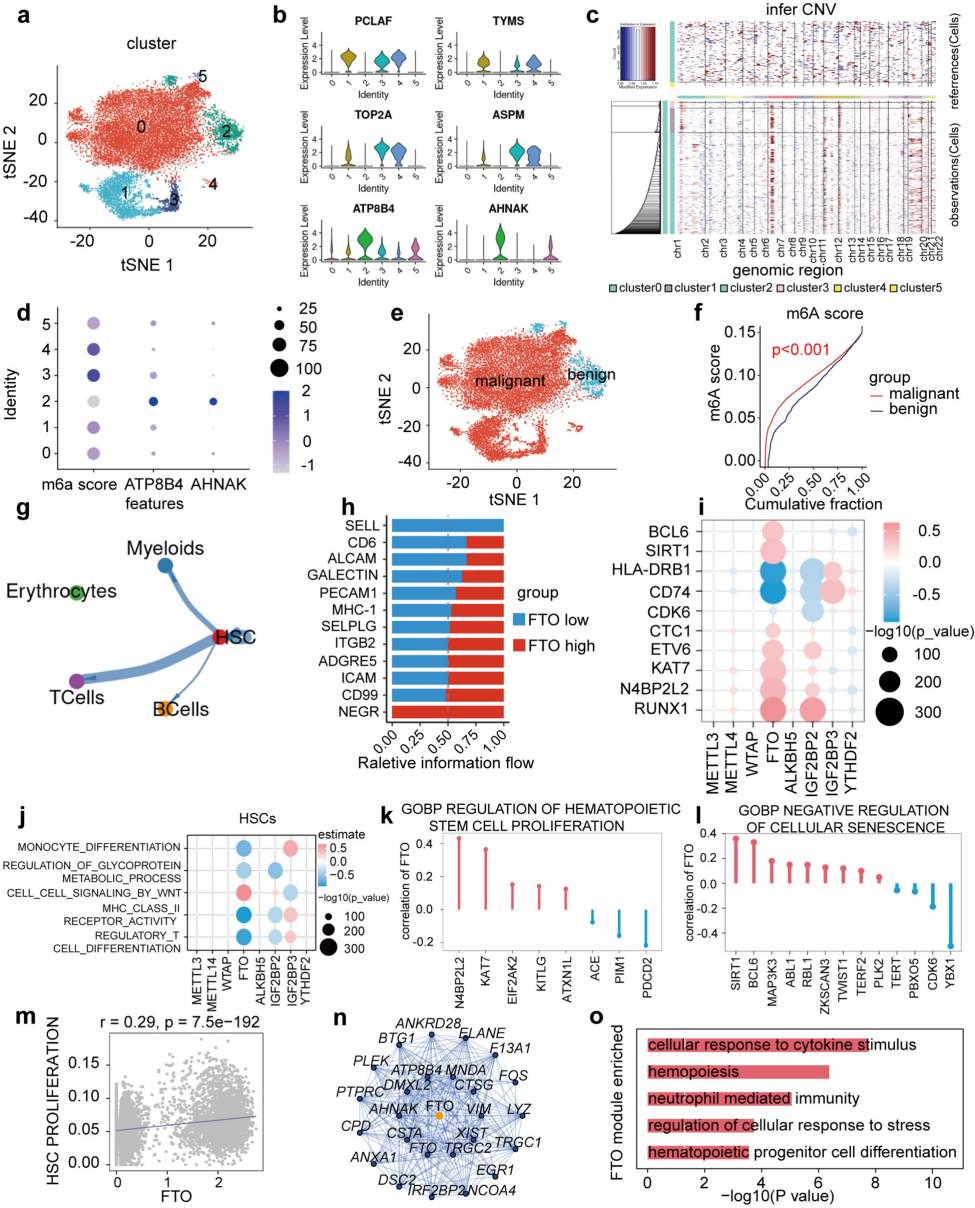
FTO upregulated caused impaired hemocyte differentiation in malignant haematopoietic stem cells (HSCs). **a** tSNE plot showing HSC subclusters. **b** Relative expression levels of the *PCLAF*,* TYMS*,* TOP2A*, *ASPM*,* ATP8B4* and *AHNAK* gene across 5 HSC subclusters. **c** Inferred Copy Number Variants (CNV) levels in each HSC cluster across 22 chromosomes. **d** The correlation between the key genes(*ATP8B4* and *AHNAK*) of HSC subclusters2 and m^6^A score signatures in HSCs. **e** tSNE plot showing the benign and malignant HSC subclusters. **f** Comparison of cumulative probability of m^6^A score signatures between benign and malignant in HSCs. (Wilcox test, *p* < 0.001). **g** and **h** According to the expression level of *FTO* in HSCs, the patients were divided into two groups; red lines represent stronger communication in *FTO* high expression group, and blue lines represent weaker communication in *FTO* high expression group (left), rank signaling networks based on the information flow or the number of interactions (right). **i** and **j** The correlation analysis between the key genes or pathways and m^6^A regulators expression in HSCs. **k** and **l** The genes of HSCs proliferation and negative regulation of cellular senescence had a significant correlation with *FTO* expression level; **m** Spearman correlation between *FTO* and HSCs proliferation. **n** Networks of weighted gene co-expression network analysis (WGCNA) module which included *FTO* in HSCs. **o** Enrichment analysis for *FTO* module by Gene Ontology (GO) enrichment

To further investigate the distinctions between the identified HSC subpopulations in the pathological process of elderly AML, we compared the expression levels of the top 20 marker genes in HSCs between normal and AML samples. All HSCs were further classified into six distinct subclusters (Fig. 3a). We found six signature genes (*PCLAF*, *TYMS*, *TOP2A*, *ASPM*, *ATP8B4* and *AHNAK*) that were significantly upregulated in HSCs. The upregulation of *PCLAF*,* TYMS*,* TOP2A*, and *ASPM* began at the malignant stage and was significantly higher in subclusters C1, C3, and C4, while the benign marker genes *ATP8B4* and *AHNAK* were significantly higher in subcluster C2 (Fig. 3b). To investigate the differences between the identified HSC subpopulations, we analyzed copy number aberrations (CNA) using InferCNV. Malignant subclusters C1, C3, and C4 exhibited copy number gains on chromosome 1, whereas subcluster C2 had low CNA levels, indicating a well-differentiated state (Fig. 3c). Further analysis using AUCell revealed that m^6^A regulators play crucial roles in regulating malignant HSCs, excluding subcluster C2 (Fig. 3d). Overall, we concluded that m^6^A regulator genes, except for *ATP8B4* and *AHNAK*, could serve as markers for AML progression.

Next, using reclustering and UMAP dimensionality reduction techniques, we observed greater heterogeneity in both benign and malignant cells in AML samples compared to control samples (Fig. 3e). To understand specific changes in m^6^A regulator expression in HSCs, A previous study assessed 23 regulators of m^6^A modification and further constructed a regulator signature, named the m^6^A score [23]. We found that the m^6^A score was upregulated in malignant HSCs (Fig. 3f), especially observed for *FTO*. To explore the mechanisms underlying HSC-*FTO* + cell communication in AML, CellChat was used to examine the interactions between *FTO* expression in HSCs and other cell types (Fig. 3g). It was observed that the *FTO* low-expression group displayed significant interactions with specific genes in other cell types in patients with AML (Fig. 3h). Moreover, we performed correlation analysis between m^6^A regulator expression levels and AML pathways. The results showed that *FTO* upregulation positively regulated the Wnt pathway but negatively regulated and correlated with monocyte differentiation, glycoprotein metabolic processes, MHC II receptor activity, and T-cell differentiation (Fig. 3j). We also revealed significant negative associations between *FTO* expression and monocyte differentiation (Fig. S3i) and significant positive associations between *FTO* expression and HSC proliferation (*r* = 0.29) (Fig. 3m). Notably, key genes involved in monocyte differentiation and HSC proliferation, such as *KAT7* and *N4BP2L2*, were positively correlated with *FTO* expression (Fig. 3k and Fig. S3g). Key regulators of cellular senescence, *BCL6* and *SIRT1*, were also positively correlated with *FTO* (Fig. 3l). Furthermore, key genes that positively regulate HSC-mediated dysfunction in elderly AML patients were positively correlated with *FTO* expression (Fig. 3i).

Additionally, we found that *WTAP* upregulation was positively associated with dysregulation in HSCs (*r* = −0.42) (Fig. S3e, f, and h). To further unravel the regulatory roles of the m^6^A regulator gene *FTO* in HSCs, we performed DEGs analysis and identified 20 genes related to *FTO* based on gene network biological validity and gene-gene interaction relevance. Weighted Gene Co-expression Network Analysis (WGCNA) showed that these genes were closely related to *FTO* expression in HSCs of AML samples (Fig. 3n). Functional enrichment analysis revealed that these genes were primarily enriched in hematopoietic cell proliferation and differentiation in AML HSCs (Fig. 3o), suggesting that *FTO* is a potentially important player in HSC regulation in AML.

### YTHDF2 cell-type-specific upregulation in erythrocytes caused negative differentiation through oxidative phosphorylation

**Fig. 4:**
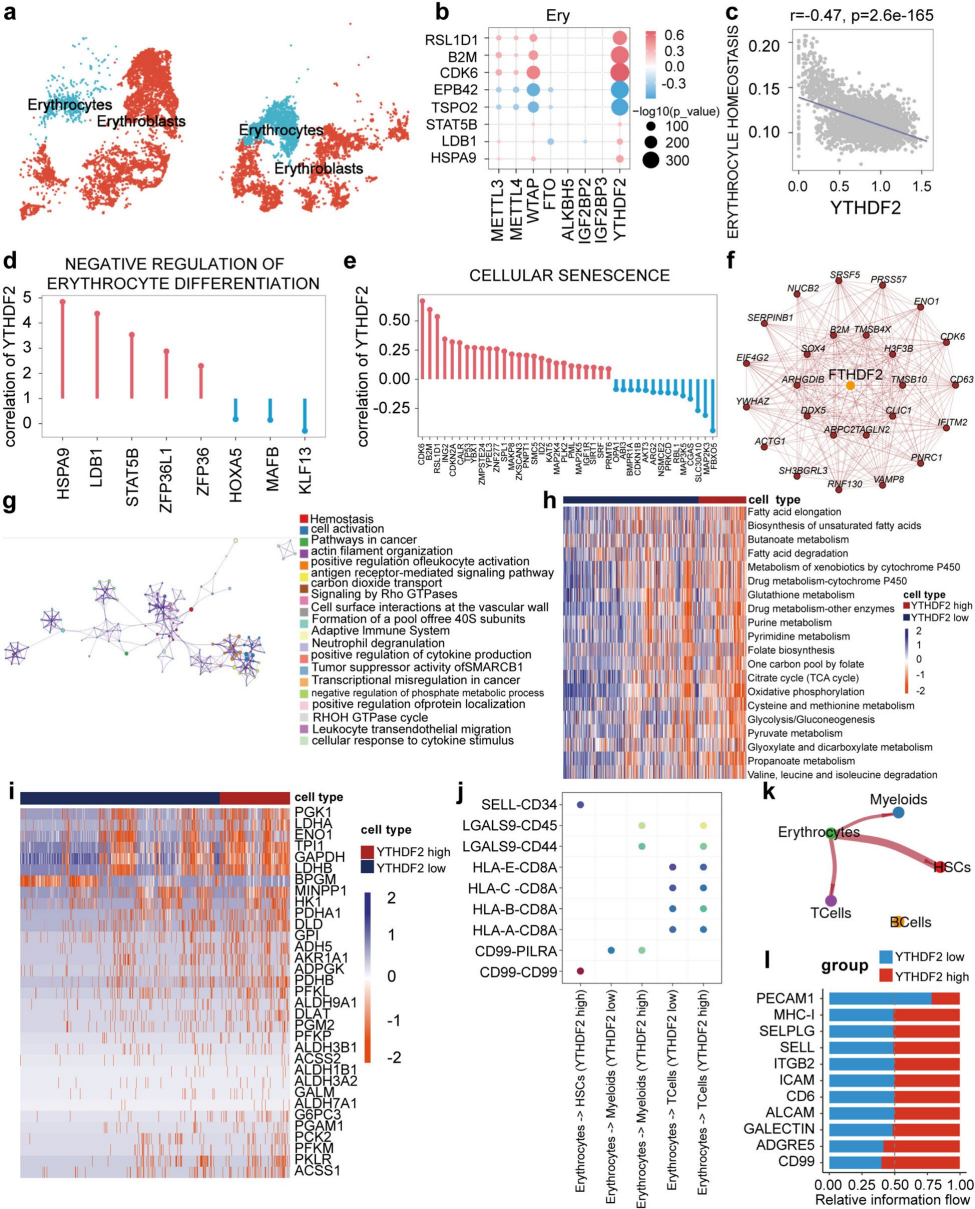
Specific upregulation of YTHDF2 in Erythrocytes resulted in negative regulation of erythrocyte differentiation. **a** Reclustering of erythrocytes and erythroblasts reveals different distribution of cells. **b** The correlation between the key genes and m^6^A regulators in erythrocytes. **c** Spearman correlation between *YTHDF2* and erythrocyte homeostasis. **d** and **e** The genes of erythrocyte differentiation and cellular senescence had a significant correlation with *YTHDF2* expression level. **f** Networks of WGCNA module which included *YTHDF2* in Erythrocytes. **g** And enrichment analysis on *YTHDF2* module by Metascape. **h** and **i** Metabolic pathway activities in Erythrocytes via *YTHDF2* high and low expression. Glycolysis in Erythrocytes via *YTHDF2* high and low expression. Statistically non-significant values are shown as blank. **j** Communication and ligand-receptor interaction between erythrocyte and neighboring cells showing in the dotplot (high *YTHDF2* versus low *YTHDF2*). **k** and **l** According to the expression level of *YTHDF2* in Erythrocytes, the patients were divided into two groups; red lines represent stronger communication in *YTHDF2* high expression group, and blue lines represent weaker communication in *YTHDF2* high expression group (left), rank signaling networks based on the information flow or the number of interactions (right)

To investigate changes in erythrocyte and erythroblast proportions between AML patients and controls, We observed substantial heterogeneity in AML samples by UMAP (Fig. 4a). We also identified *YTHDF2* as a key gene in regulating erythrocyte processes in elderly AML patients. We determined the expression of *YTHDF2* was higher in Erythrocytes by MetaCell (Fig. 4b). To explore the cell-type-specific expression pattern of *YTHDF2* dysregulation-triggered differentiation dysfunction, correlation showed that *YTHDF2* negatively regulated erythrocyte differentiation, cellular senescence (Fig. 4d and e), and erythrocyte homeostasis (*r*=−0.47) (Fig. 4c), suggesting that *YTHDF2* cell-type-specific upregulation caused dysfunction of Erythrocytes. Key regulators of the negative regulation of erythrocyte differentiation, including *HSPA9*, *LDB1*, and *STAT5B*, were positively correlated with *YTHDF2* expression (Fig. 4d). Key regulators of cellular senescence, were also positively correlated (Fig. 4e). Next, WGCNA was also used to identify the role of *YTHDF2* in erythrocytes from elderly AML patients (Fig. 4f). Metascape analysis showed these genes were linked to leukocyte activation, cancer pathways, and phosphate metabolism (Fig. 4g). This suggests *YTHDF2* upregulation causes dysfunctional erythrocyte differentiation and affects leukocyte activation in elderly AML patients.

To quantify the activity of metabolic pathways affected by *YTHDF2* upregulation in erythrocytes, we used a pathway activity heatmap showing the correlation. We found that *YTHDF2* upregulation positively regulated oxidative phosphorylation, glycolysis/gluconeogenesis, folate biosynthesis, and glycosaminoglycan biosynthesis (Fig. 4h). Notably, key genes involved in glycolysis/gluconeogenesis, including *PGK1*, *DHA*, *ENO1*, *TPI1*, *GAPDH*, and *LDHB*, were upregulated by *YTHDF2* upregulation (Fig. 4i). To explore the mechanisms underlying erythrocyte-*YTHDF2* + cell communication in AML, CellChat was used to examine the interaction between *YTHDF2* expression in Erythrocytes and other cells (Fig. 4j and k), and it was observed that the *YTHDF2* high-expression group displayed significant interactions with specific genes (Fig. 4l).

### FTO cell-type-specific upregulation in Myeloids and TCells caused negative regulation of haematopoietic progenitor cell and T-cell-mediated immunity

**Fig. 5:**
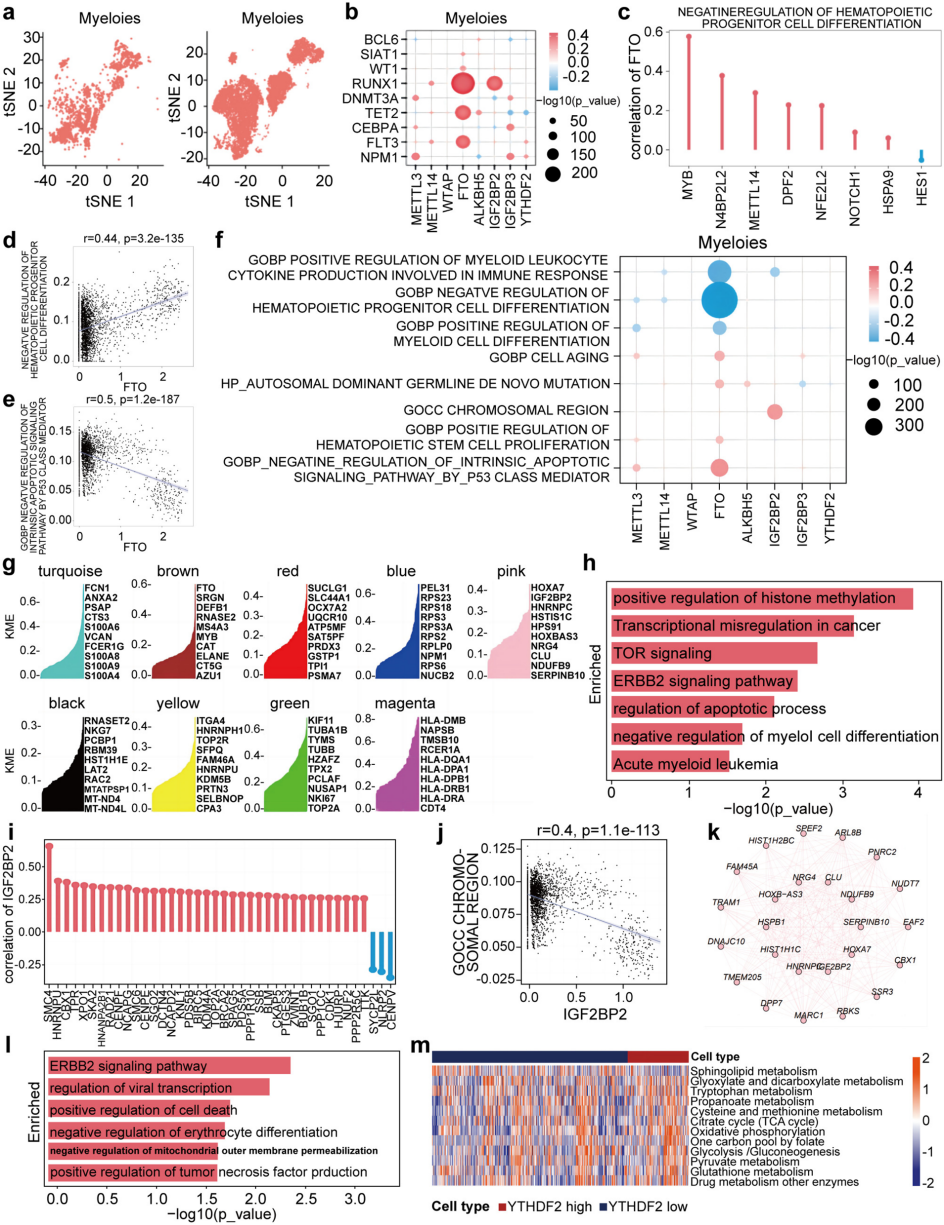
FTO and IGF2BP2 promoted the increase of pathological myeloid differentiation. **a** Reclustering of Myeloids reveals different distributions of malignant and normal cells. **b** The correlation between the key genes and m^6^A regulators in Myeloids. **c** The genes of negative regulation of haematopoietic progenitor cell differentiation had a significant correlation with *FTO* expression level. **d** and **e** Spearman correlation between *FTO* and negative regulation of haematopoietic progenitor cell differentiation, negative regulation of apoptosis pathway. **f** The correlation analysis between the key pathways and m^6^A regulators expression in Myeloids. **g** WGCNA identifies brown modules with correlated gene expression patterns in Myeloids of AML patients. **h** Enrichment analysis for *FTO* module by GO enrichment. **i** The genes of chromosomal region had a significant correlation with *IGF2BP2* expression level. **j** Spearman correlation between *IGF2BP2* and chromosomal region. **k** Networks of PINK module which included *IGF2BP2* in Myeloids. **l** And enrichment analysis for PINK module by GO enrichment. **m** Metabolic pathway activities in Myeloids via *IGF2BP2* high and low expression

To explore the heterogeneity in myeloid cells in elderly AML patients, we identified patterns using UMAP (Fig. 5a). To determine the causes of dysregulation in myeloid cells by m^6^A regulators, we performed correlation analysis between m^6^A expression and AML pathways. We found that key genes positively regulating myeloid-mediated dysfunction in elderly AML patients were positively correlated with *FTO* expression (Fig. 5b). The results showed that *FTO* upregulation positively regulated HSC abnormal proliferation and cell aging, while negatively regulating and correlating with pathways such as myeloid cell differentiation, HSC differentiation, apoptotic signaling pathways, and immune response in AML (Fig. 5f). Specifically, *FTO* upregulation negatively regulated hematopoietic progenitor cell differentiation (*r* = 0.44) (Fig. 5d) and myeloid differentiation and the p53 pathway (*r* = −0.5) (Fig. 5e). The expression of key genes involved in negative hematopoietic progenitor differentiation was positively correlated with *FTO* (Fig. 5c).

To elucidate cell-specific ligand-receptor pairs between myeloid cells and other cell types, CellChat analysis indicated that *FTO* played a regulatory role (Fig. S3j). To identify the function of *FTO* upregulation in myeloid cells, we divided the WGCNA results into nine modules (Fig. 5g). *FTO* was identified in the brown module in myeloid cells. Functional enrichment analysis revealed that this module was involved in the regulation of the apoptotic process and negative regulation of myeloid cell differentiation in AML samples (Fig. 5h). Next, we found that *IGF2BP2* upregulation induced dysfunctional chromosomal regions (*r* = 0.4) involved in myeloid cells in AML (Fig. 5i and j). WGCNA showed that 20 genes were closely related to *IGF2BP2* in AML myeloid cells (Fig. 5k). Functional enrichment analysis indicated that *IGF2BP2* upregulation negatively regulated erythroid differentiation in myeloid cells in AML samples (Fig. 5l). The metabolic pathways dysregulated by *IGF2BP2* upregulation in myeloid cells showed that *IGF2BP2* upregulation was also positively regulated by oxidative phosphorylation and glycolysis/gluconeogenesis (Fig. 5m). In addition, we determined that *FTO* was a key gene in the positive regulation of granulocyte-monocyte progenitor (GMP)-mediated processes (Fig. S3k and Fig. S3l).

To examine the expression pattern of *FTO* in T cells, we found that *FTO* upregulation positively correlated with cellular senescence (*r* = 0.41) but negatively affected T-cell-mediated immunity (*r* = −0.4). WGCNA identified biological functions involved in the negative regulation of immune cell differentiation, suggesting that *FTO* upregulation leads to dysfunctional immune responses in T cells. We also identified ligand-target interactions between T cells and HSCs, highlighting the role of HMGB1-ligands in their communication using NicheNet (Fig. S4).

### Heterogeneity transitional trajectory and dysfunctional differentiation of haemocyte regulatory networks in AML

**Fig. 6:**
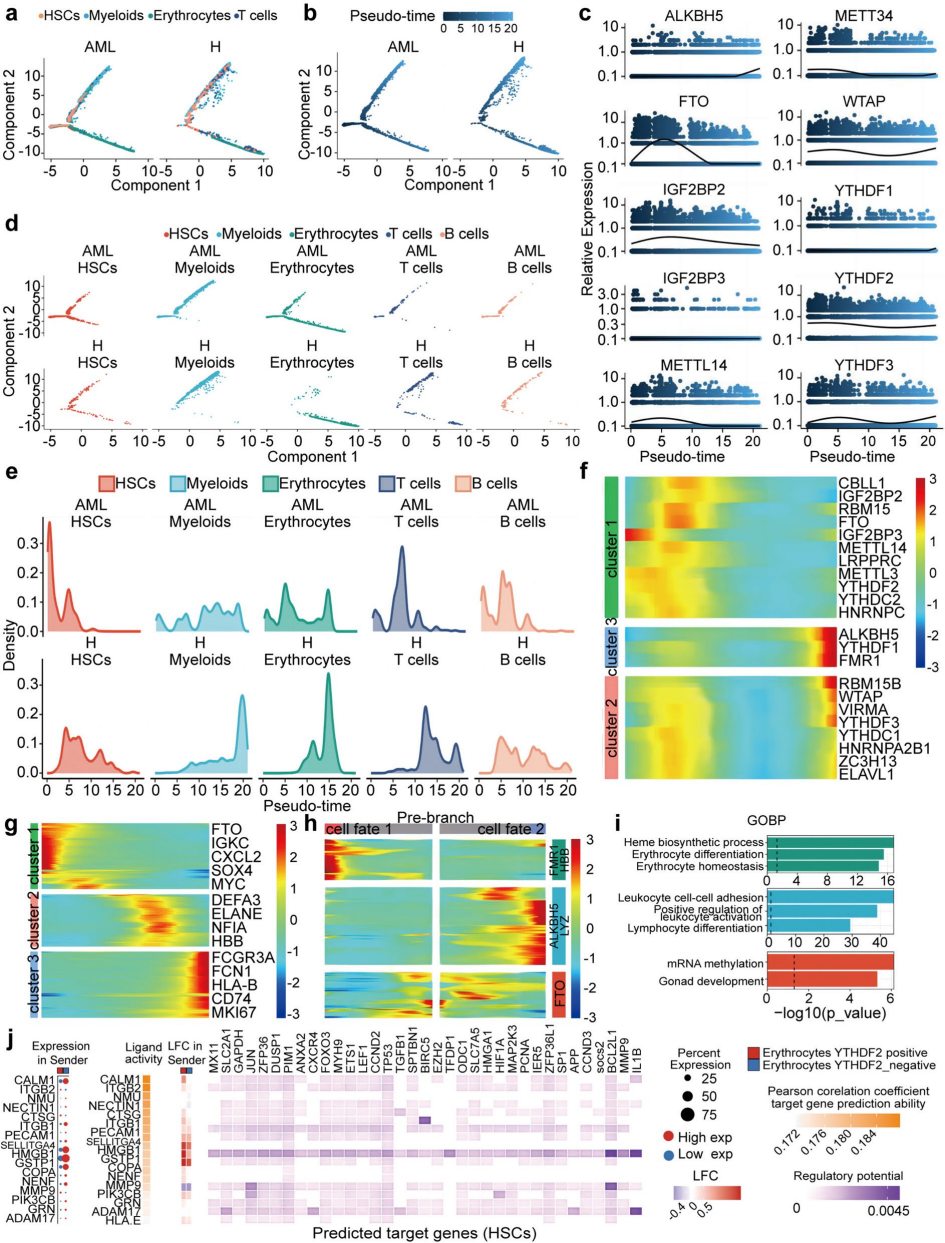
m^6^A regulators dysregulated cytopathologic differentiation in diverse cell types. **a** Pseudo-time trajectory of diverse cell type, **b** and Pseudo-time trajectory of cell of AML with gene expression profiles inferred by Monocle 2. Each point corresponds to a single cell. **c** Fitted curves showing dynamic expression changes in representative m^6^A genes in each gene group based on AML progressing time. **d** Pseudotime analysis of each cell type. HSCs, Myeloids, Erythrocytes, TCells and BCells were divided into relatively malignant (Pseudotime < 5) and benign (Pseudotime ≥ 5) groups. **e** The proportions of HSCs, Myeloids, Erythrocytes, TCells and BCells; pseudo-time was shown on the horizontal axis. **f** Heatmap of m^6^A regulator expression profiles based on pseudo-time trajectory of 3 cluster cells of AML. **g** Heatmap of key genes of 3 cluster cells profiles based on pseudo-time is indicated on the horizontal axis. **h** Heatmap representing the smoothed expression of pseudo-time-dependent genes along pseudo-timeline of AML cell fate branch; Pseudo-time-dependent genes were grouped into three clusters according to different expression patterns, and the m^6^A regulators included in each module were annotated. **i** Bar plot of key genes of HSCs module, Myeloids module, Erythrocytes module enriched pathways. **j** NicheNet was used to analyze the expression of ligands and receptors to identify the intercellular communication patterns between Erythrocytes and HSCs

To elucidate the mechanism of the transition from benign to malignant HSCs, pseudotime analysis was performed on five cell clusters in AML samples (Fig. 6a). For HSCs, a large proportion of cells were in an initial state of differentiation from benign to malignant HSCs (Fig. 6b). Notably, subcluster 1 cells represented malignant HSCs with impaired differentiation, and there was an increase in the number of subcluster 1 cells within the HSC population (Fig. 6d). Additionally, *FTO* had higher expression levels during the initial stage of disease (Fig. 6c). Pseudotime analysis predicted that the majority of malignant cells originated from initial HSCs (Fig. 6e), and that the majority of malignant HSCs originating from subcluster 1 were dysregulated by *FTO*, with a few benign cells differentiating into other cell clusters (Fig. 6f and g). Gene Ontology (GO) enrichment analysis of upregulated genes in the initial stage (Fig. 6h) revealed that subcluster genes were involved in pathways such as mRNA methylation (Fig. 6i). Overall, these results contribute to the understanding of the hierarchical structure and heterogeneity of malignant HSCs dysregulated by *FTO* in AML.

Similarly, pseudotime analysis was performed on erythrocytes, and subcluster identification was conducted to investigate the transition of *YTHDF2*-expressing erythrocyte differentiation (Fig. 6c). *YTHDF2* had higher expression in subcluster 1 compared to other subclusters (Fig. 6f), and was primarily enriched in functions related to erythrocyte differentiation and erythrocyte homeostasis (Fig. 6i).

Pseudotime analysis was also conducted on myeloid cells (Fig. 6a and b). *FTO* and *IGF2BP2* had higher expression levels during the intermediate stage (Fig. 6c). The AML group had a higher number of fate 2 cells compared to the control group (Fig. 6d and h), which were enriched in the positive regulation of leukocyte activation (Fig. 6i). Moreover, we found that *FTO*, *YTHDF2* and *IGF2BP2* clustered together in subcluster 1 (Fig. 6f), and common oncogenes in AML, such as *IGKC*, *CXCL2*, *SOX4* and *MYC* (Fig. 6g).

To elucidate cell-specific ligand receptors among diverse cell types across m^6^A regulators in AML, intercellular communication between Erythrocytes and HSCs was examined using NicheNet. Ligand activity prediction was performed to identify the most likely *YTHDF2*-regulating ligands. Notably, one of the top-ranked ligands was *HMGB1*. Subsequently, we assessed the extent to which *HMGB1*-ligands may regulate aberrant erythrocyte differentiation by the 20 top-ranked *HMGB1*-ligands can predict dedifferentiation-related genes. Finally, we identified the interactions between *HMGB1* ligands and erythrocyte differentiation genes in AML (Fig. 6j). Overall, we identified the potential active ligand-target links that played specific dedifferentiation regulatory roles of *HMGB1*-ligands that were dysregulated by *YTHDF2*.

Additionally, NicheNet analysis was performed to identify ligand-target links between Myeloids and others. Similarly, NichNet was used to identify potential active ligand–target links between HSCs and Myeloids, ligand–target links between TCells and Myeloids. Taken together, results suggest the diverse potential of m^6^A regulators in the regulatory function of specific types of AML (Fig. S5).

### The validation of mechanism for m6A regulator cell-type-specific upregulation in diverse cell types of AML by scRNA-Seq data, and experiment test in vitro of cell function and oxidative phosphorylation

To verify cellular diversity and m^6^A regulator expression in AML, we analyzed 17 samples with scRNA-Seq (GSE235923). After quality control, 35,203 cells were retained, including HSCs, myeloid cells, erythrocytes, T cells, B cells, and plasma cells (Fig. S6a). Several m^6^A regulators, showed high expression levels and high proportion in Erythrocytes, HSCs and myeloid cells. Reclustered the expression of *YTHDF2* and *IGF2BP2* (Fig. S6b, c, d and e), Calculating an m^6^A score in diverse cell types, suggesting abnormal immune cell differentiation (Fig. S6f).

To validate the cell-type-specific mechanisms of m^6^A regulators in AML, *FTO* in HSCs were defined as ‘malignant HSC’ (MHSC) signature. *FTO* and MHSC showed a positive correlation. *YTHDF2* in erythrocytes were defined as ‘malignant erythrocytes’ (MERY) signature. *YTHDF2* and MERY showed a positive correlation. *FTO* in myeloid cells were defined as ‘malignant myeloid cells’ (MMYE) signature. *FTO* and MMYE showed a positive correlation. *IGF2BP2* and *SMC4* expression showed a positive correlation. (Fig. S6g, h, i and j). The results demonstrated that *FTO*, *YTHDF2*, and *IGF2BP2* play important cell-specific regulatory roles in AML.

To verify cell-type-specific m^6^A regulators in AML, we used MetaCell and WGCNA. Results showed 25 genes related to *YTHDF2* in erythrocytes, indicated these genes affect immune response. Overall, through another datasets of AML, we proved *FTO* was specifically upregulated in HSCs. *YTHDF2* was upregulated in erythrocytes. *FTO* and *IGF2BP2* were upregulated in myeloid cells (Fig. S6k and Fig. S6l).

**Fig. 7:**
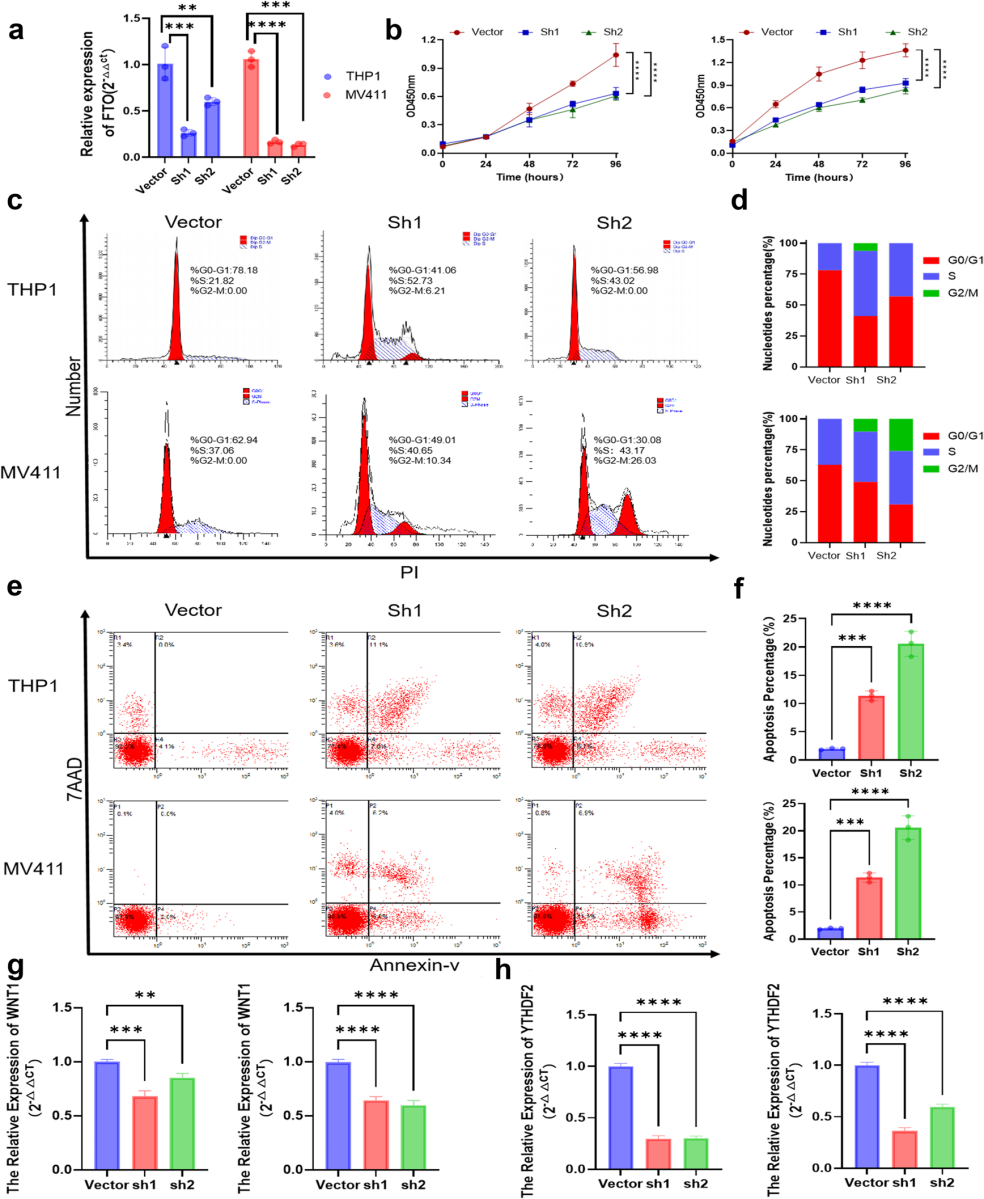
FTO regulated THP-1 and MV411 cells proliferation and oxidative phosphorylation, while inhibited cell cycle arrest and apoptosis. **a** Quantitative Real-Time Polymerase Chain Reaction (qRT–PCR) analysis of *FTO* expression in THP-1 and MV411 cells transfected with sh-NC and sh-*FTO* expression vector. **b** The growth curves of cells transfected with indicated vectors were evaluated by Cell Counting Kit-8 (CCK-8) assays. **c** and **d** The cell cycle progression was analyzed by flow cytometer after being transfected with indicated plasmids. **e** and **f** Apoptosis rate was analyzed by flow cytometer after being transfected with sh-NC and sh-*FTO* expression vector. **g** and **h** *WNT1* and *YTHDF2* expression in THP-1 and MV411 cells transfected with sh-NC and sh-*FTO* expression vector, ** and *** represent *p* < 0.01 and *p* < 0.001, respectively (THP-1 cell on the left and MV411 cell on the right of the bar graphs)

To further verify the role of *FTO* and its downstream-related mechanisms in AML cells in vitro, we constructed shRNA-*FTO*−1(sh1) and shRNA-*FTO*−2(sh2) plasmids for transfection into the THP1 and MV411 cell lines, to measure the cell proliferation, migration and apoptosis. The efficiency of silencing was verified by Quantitative Real-Time Polymerase Chain Reaction (qRT–PCR) after the plasmid transfection is complete (Fig. 7a). To measure the cell proliferation, Results from the Cell Counting Kit-8 (CCK-8) assay demonstrated that the silencing of *FTO* effectively inhibits the survival rate of THP-1 and MV411 cells (*p <* 0.001) (Fig. 7b). To begin exploring the mechanisms by which *FTO* might regulate cell cycle progression in AML cells, we performed cell cycle analysis of THP-1 and MV411 cells by using flow cytometry, cell cycle analysis revealed that silencing *FTO* led to a decreased percentage of THP-1 and MV411 cells in G0-G1 phase and an increased percentage of cells in S phase compared to the control group (Fig. 7c and d). To investigate the apoptotic effects of *FTO* on AML cells, We used an apoptosis kit to assess the apoptotic effects of *FTO* on THP-1 and MV411 cells by flow cytometry. *FTO* flow cytometer analysis using Annexin V/PI double staining showed a significant increase in the apoptosis rate of cells transfected with sh-*FTO* (*p <* 0.001) (Fig. 7e and f). Additionally, to determine the effects of *FTO* on the invasion of AML cells, transwell assays were conducted in THP-1 and MV411 cells after knockdown of *FTO*. The results showed that downregulation of *FTO* significantly attenuated THP-1 and MV411 cells migration and invasion (*p <* 0.001) (Fig. S7a and Fig. S7b). These results suggested that *FTO* exhibited important regulation of cell proliferation, cell cycle progression, apoptosis, and cell invasion in THP-1 and MV411 cells.

To verify the association between *FTO* and oxidative stress in vitro experiment in AML, the expression level of reactive oxygen species (ROS) in THP-1 and MV411 cells after knockdown of *FTO* was examined through the ROS assay kit. The results showed that ROS levels were attenuated in both THP-1 and MV411 cells after knockdown of *FTO* (Fig. S7c). To evaluate the regulatory role of the *FTO*/WNT pathway and *YTHDF2* in oxidative phosphorylation, we performed the PCR to analyse the expression of *WNT1* and *YTHDF2* after knockdown of *FTO*. The results showed that knockdown of *FTO* resulted in lower expression of *WNT1* and *YTHDF2* in THP1 and MV411 cells than in the non-knockdown group (*p <* 0.001) (Fig. 7g and h). The results of RNA quantification revealed that knocked down *FTO* expression in THP-1 and MV411 cells was associated with m^6^A levels. Overall, *FTO* exerts significant regulatory effects on the proliferation, invasion, apoptosis, oxidative phosphorylation, m^6^A regulation and downstream tumor-related signaling pathways in AML cells.

## Discussion

Although considerable progress has been made to alter proliferation/apoptosis, signal transduction, epigenetic modification and immunity in AML, patients still have a poor prognosis and a high mortality rate due to the complexity of the pathogenesis of elderly AML [15, 24–27]. Recently, investigations into epigenetic modification of RNA at the post-transcriptional level have attracted widespread attention, and m^6^A modification, which is the most common RNA modification, has been extensively studied in the occurrence and development of AML [28].

ScRNA-seq was used to identify differences in m^6^A modification between AML patients and healthy controls. Our findings showed that m^6^A regulation varies among different cell types in elderly AML patients. *FTO* upregulation in HSCs, Myeloids, and T-cells inhibited their differentiation through the WNT pathway. *YTHDF2* was elevated in erythrocytes, negatively regulating differentiation via oxidative phmyeloidsosphorylation and causing leukocyte activation. *IGF2BP2* was upregulated in, leading to dysfunctional chromosomal regions and dysregulated oxidative phosphorylation. Further analysis with CellChat confirmed changes in ligand-receptor interactions mediated by m^6^A regulators, activating the HMGB1 pathway and promoting AML progression. In vitro experiments using shRNA targeting *FTO* demonstrated inhibition of cell proliferation, migration, and oxidative phosphorylation, as well as induction of cell cycle arrest and apoptosis.

*FTO* is an RNA m^6^A demethylase that belongs to the Alkb protein family [29]. Previous studies have shown that *FTO* is abnormally upregulated in certain subtypes of AML, such as t (11q23)/*MLL* rearrangement, t (15; 17), *FLT3-ITD*, and/or *NPM1*-mutated AML [30]. Our results further demonstrated that *FTO* was highly expressed in HSCs and increased with the differentiation of HSCs. In addition, we explained the function of *FTO* in HSCs. *FTO* was positively correlated with HSC proliferation genes (*KAT7* and *N4BP2L2*) and negatively correlated with monocyte differentiation genes (*MYC*, *IFI16*, *HLA-DRB1*, and *CD74*), suggesting that *FTO* can promote HSC proliferation and inhibit HSC monocyte differentiation. Li et al. previously reported that *FTO* downregulates the expression of *ASB2* and *RARA* by reducing their m^6^A levels, thereby promoting the survival/proliferation of AML cells and inhibiting differentiation and apoptosis [30]. In addition, Li et al. demonstrated that this axis can inhibit the differentiation of AML cells induced by *ATRA* [30]. Subsequently, Su et al. demonstrated that *FTO* can also promote the proliferation of leukaemia cells through *FTO*/m^6^A/*MYC*/*CEBPA* signaling [11]. We similarly observed positive correlations between *FTO* and *N4BP2L2* and *RUNX1*, indicating that *FTO* was also involved in the *WNT* signaling pathway in HSCs. Previous studies have demonstrated that in multiple myeloma, *IDH2* enhances the expression of *WNT7B* and activates the *WNT* signaling pathway by targeting the m^6^A demethylase *FTO*, thereby promoting the occurrence and development of multiple myeloma [31]. The importance of *FTO* in regulating *WNT* signalling pathways has also been reported in solid tumors, which was consistent with our research results [32–34]. Intriguingly, our study revealed that *FTO* was positively correlated with *BCL6* and *SIRT1*, suggesting its potential involvement in regulating cellular senescence. Li et al. substantiated this point by demonstrating that *FTO* induces endothelial cellular senescence [35].

We also proved that *FTO* was highly expressed in Myeloids, and inhibited haematopoietic progenitor cell differentiation, myeloid differentiation, *p53* signaling pathway. Our observations revealed a negative correlation between *FTO* and the *p53* signaling pathway, suggesting that *FTO* may regulate the proliferation and differentiation of hematopoietic stem/progenitor cells through this pathway. Furthermore, *FTO* exhibited an impact on immune responses in Myeloids, influencing the therapeutic resistance of AML. The use of small-molecule inhibitors targeting *FTO* affected the expression levels of downstream genes such as *MYC*, *CEBPA*, *RARA*, and *ASB2*, thereby exerting anti-leukaemia effects [36]. Moreover, *FTO* can also inhibit the expression of immune checkpoint genes, especially *LILRB4*, significantly weakening the self-renewal and reprogramming immune response of leukaemia stem cells/initiating cells, which has been proven to be an effective treatment strategy for AML [36, 37]. Additionally, the upregulation of *IGF2BP2* in Myeloids induced a dysfunctional chromosomal region, which is influenced by oxidative phosphorylation and glycolysis/gluconeogenesis.

In addition, we serendipitously found significant differences in the Erythrocytes between patients and healthy individuals during cluster analysis, especially with a significant increase in erythroblasts in patients. Previous studies have shown that erythroid progenitor cells derived from tumor hosts lose their erythrocyte potential and differentiate into myeloid cells, named erythroid-differentiated myeloid cells (EDMCs) [18, 38]. Our study suggested that the binding protein *YTHDF2* of m^6^A was involved in the proliferation and differentiation of erythrocytes, regulating erythrocyte homeostasis through oxidative phosphorylation, and *YTHDF2* was highly expressed in Erythrocytes rather than in other types of cells. *YTHDF2* is significantly more highly expressed in AML samples with multiple cytogenetic abnormalities and is essential for AML cell survival and leukaemia cell implantation, as reported by Paris et al. [12]. Moreover, *YTHDF2* reduces the half-life of various m^6^A transcripts, which are crucial to the functional maintenance of LSCs [12]. Paris et al. also believed that *YTHDF2* was not important for steady haematopoiesis, while Mapperley et al. reached the opposite conclusion [12, 39]. It is necessary to further clarify the role of *YTHDF2* in HSCs and its function in regulating the deterioration of HSCs in the future.

To further validate the function of *FTO*, we conducted *FTO* silencing experiments in THP-1 cells. Our findings suggest that silencing *FTO* suppresses proliferation, induces cell cycle arrest, and promotes apoptosis in THP-1. These results, combined with previous studies, underscore the pro-leukemic role of *FTO* in vitro [11, 30, 40].

To explore the cell-specific ligand-receptor pairs among diverse cell types, we used NicheNet to examine intercellular communication, and the results showed that *HMGB1* ligands dysregulated by *YTHDF2* were related to erythrocyte differentiation genes in AML. *HMGB1* ligands are also involved in the communication between Myeloids/TCells and other cell types. *HMGB1* ligands may activate proinflammatory pathways mediated by *IL1*β in the HSC-to-myeloid cell transition. This finding is consistent with recent observations that *HMGB1* is highly expressed in BM mononuclear cells of AML patients, which affects the activity of the downstream gene *TGFBI* and the expression levels of *TNF-α*, *IL-1β* and *SCF*, blocking AML cell proliferation and myeloid cell differentiation [41–43].

The study has some limitations. Our research primarily focused on how AML affects blood cell differentiation and the role of m^6^A regulators in HSCs, myeloid cells, and T-cells, specifically how m^6^A regulators inhibits their differentiation via various signaling pathway. However, we did not cover the full systemic impact of the disease or conduct animal experiments to validate the impact of cell-cell ligand receptor interactions on AML progression. Additionally, we did not study cell damage caused by AML progression at the spatiotemporal level in diverse cell types, which was due to the small sample size. Moving forward, we plan to investigate more m^6^A regulators and their effects on cell types involved in AML. We will also perform animal experiments to validate the impact of cell-cell interactions and investigate the full-body effects of AML using multi-omics approaches and clinical data analysis.

In conclusion, we showed that the m^6^A score could be used to assess patients’ clinicopathological subtype features of diverse cell types, including stages of CNV, tumor differentiation levels, and molecular subtypes. Detailed relationships between m^6^A score and clinicopathological features of AML were revealed in our study. We could also predict the efficacy of chemotherapy and patient clinical response to immunotherapy using m^6^A regulators, *FTO* was upregulated in HSCs, Myeloids and TCells, which inhibited the differentiation of diverse cell types through the *WNT* pathway. *YTHDF2* upregulation in Erythrocytes induced the negative regulation of differentiation through oxidative phosphorylation, leading to leukocyte activation. m^6^A regulators induced aberrant cell-cell communication in haemocytes and mediated ligand-receptor interactions across diverse cell types. Our study provided a new avenue for improving patient clinical responses to immunotherapy, identifying distinct tumor immune phenotypes, and promoting personalized AML immunotherapy. Moving forward, we plan to investigate additional m^6^A regulators and their effects on various cell types involved in AML. Through these endeavors, we aim to uncover new therapeutic targets and facilitate the development of more comprehensive treatment plans for AML patients. Overall, the findings of the present study provide a novel approach for developing drug targets for patients with AML.

## Materials and methods

### Dataset source and preprocessing

Data for three elderly AML patients and two healthy donors enrolled in this study were sourced from the Department of Hematology at the Third Hospital of Shanxi Medical University between January 2021 and March 2022. The study protocol was approved by the Ethics Committee of the Third Hospital of Shanxi Medical University, China (Approval No.: SBQKL-2021-051). Bioinformatics analysis was conducted on single-cell RNA sequencing (scRNA-seq) data derived from bone marrow (BM) blood cell samples collected from patients prior to their receiving any treatment. ScRNA-seq datasets of AML BM cells were obtained from the Gene Expression Omnibus (GEO) database under accession number GSE235923 (accessible at www.ncbi.nlm.nih.gov/GEO). Additionally, bulk RNA-seq datasets for both AML BM cells and healthy donor BM cells were also retrieved from the GEO database (accession number GSE116616). Detailed information regarding all samples, including the time elapsed since diagnosis, sex, age, and mutation profiles, is provided in Supplementary Tables 1 and Supplementary Table 3.

The BD Rhapsody Analysis pipeline and the BD AbSeq Ab-Oligos Panel were employed to quantify FASTQ data for the single-cell RNA sequencing (scRNA-seq) analysis of the samples collected for this study. The quantified data were aligned against the human reference genome (hg19) to generate a matrix of raw counts. As part of our quality control measures, we filtered out outliers using a threshold of three times the Median Absolute Deviation (MAD) above or below the median. To correct for differences in gene expression levels among cells, we applied the LogNormalize method for global scaling normalization, standardizing both the characteristic and total expression per cell.

### Analysis of scRNA-seq data

The expression data from the control and AML samples were integrated using the FindIntegrationAnchors and IntegrateData functions within the Seurat package. Normalization was performed using the ScaleData function in Seurat, ensuring that each gene was weighted equally, with a mean of zero and a variance of one. To reduce computational complexity and noise, principal component analysis (PCA) was utilized for initial dimensionality reduction. K-nearest neighbors (K-NN) graphs were constructed using the FindNeighbors function based on the Euclidean distances in the PCA space. Cell clustering was performed using the Louvain algorithm. Finally, the annotated information for each cell was visualized using Uniform Manifold Approximation and Projection (UMAP).

### Pseudotime trajectory analysis of m6A mRNA regulators in AML cells

To investigate the relationship between cell pseudotime trajectories and m6A regulators, we utilized the Monocle R package to analyze single-cell RNA data across all cell types. Highly variable genes were identified based on differential expression between two specific cell types. Dimensionality reduction was performed using the DDRTree method. Subsequently, we employed the plot_pseudotime_heatmap function to visualize heatmaps depicting the dynamic expression of m^6^A regulators along the pseudotime trajectories of different pulmonary cell types in AML patients.

### Construction of metacell to conduct DEGs and correlation analysis

The concrete details can be found in supplementary information.

### Cell culture and transfection

THP-1 cells and MV411 cells (obtained from ATCC) were cultured in RPMI-1640 medium (Gibco) supplemented with 10% fetal bovine serum (FBS) and 1% penicillin-streptomycin (Gibco). 293T cells (donated by the Chinese Academy of Medical Sciences) were maintained in DMEM medium (Gibco) supplemented with 10% FBS. All cell lines used in this study were authenticated by short tandem repeat (STR) analysis to confirm their identity and were found to be mycoplasma-free. All cell cultures were incubated in a constant temperature incubator (Thermo Scientific) at 37 °C in an atmosphere of 5% CO_2_. *FTO* short hairpin RNAs (shRNAs) were cloned into the pGPU6/GFP/Neo lentiviral vector (synthesized by GenePharma). All target sequences for the shRNAs are listed in Supplementary Table 2. Lentiviral particles were produced in 293T cells and used to infect THP-1 cells. The efficiency of gene transfer into THP-1 cells was assessed by detecting green fluorescent protein (GFP) using laser confocal microscopy (Olympus) and a flow cytometer (Beckman).

### Quantitative polymerase chain reaction (qPCR)

Total RNA was extracted from cell samples using Trizol reagent (Ambion, USA). The mRNA was then reverse transcribed into cDNA using the cDNA Synthesis SuperMix (TransGen Biotech, Beijing). PCR amplification was conducted using a PCR machine (Bioer Technology, GeneMax Tc-s-B, China) using the following cycling parameters: an initial denaturation at 95 ℃ for 5 min, followed by 40 cycles of denaturation at 95 ℃ for 10 s, and annealing at 60 ℃ for 30 s. Gene expression levels were quantified using the ΔΔCt method. The experiments were performed in triplicates to ensure reproducibility. The primers used for RT-qPCR were as follows:

- GAPDH forward: 5’-GCTCTCTGCTCCTCCTGTTC-3’;
- GAPDH reverse: 5’-ACGACCAAATCCGTTGACTC-3’;
- *FTO* forward:5′-AАCАСCAGGCTCTTTACGGTC-3′;
- *FTO* reverse:5′-TGTCCGTTGTAGGATGААССС−3′.

### Cell proliferation, apoptosis, cell cycle, and cell migration assays

A cell viability assay was performed using the CCK-8 Assay Kit (DOJINDO). Approximately 5,000 cells were seeded per well in a 96-well plate. Apoptosis in THP-1 cells was detected using the Annexin V PE/7-AAD Apoptosis Assay Kit (Bioss) according to the manufacturer’s instructions. Additionally, the cell cycle was analyzed using the Cell Cycle Analysis Kit (Bioss) and evaluated with ModFit software. For cell migration assays, Transwell plates (Corning) were used. Cells were initially incubated in serum-free medium for 24 h. Subsequently, 1 × 10^5^ cells were suspended in 200 µL of serum-free RPMI-1640 medium and added to the upper chamber. Meanwhile, 1 mL of medium containing 20% FBS was placed in the lower chamber. After 24 h of incubation, the migrated cells were photographed and manually counted.

### Statistical analysis

All statistical analyses were conducted using R software (version 4.0). Student’s *t* test was used to evaluate the statistical significance of differences between two different groups. A *p* value < 0.05 was considered statistically significant.

## Supplementary Information

Supplementary Material 1.

## Data Availability

Single-cell RNA sequencing gene expression data generated in this study has been deposited in the ArrayExpress database with accession of E-MTAB-19818. (https://www.ebi.ac.uk/fg/annotare/edit/19818/#DESIGN:PROTOCOLS). Raw sequencing data and processed data are available through the NCBI Gene Expression Omnibus (GEO) under accession number “GSE116616” and “GSE235923”. All data needed to evaluate the conclusions in the paper are present in the paper and/or the Supplementary Materials.

## Abbreviations

HSCs: Haematopoietic stem cells
AML: Acute myeloid leukaemia
m^6^A: N6-methyladenosine
scRNA-seq: single-cell RNA sequencing
shRNA: short hairpin RNA
cCR: composite complete remission
OS: Overall survival
LSCs: Leukaemia stem cells
LICs: Leukaemia initiating cells
BM: Bone marrow
GEO: Gene Expression Omnibus
MAD: Median Absolute Deviation
PCA: Principal component analysis
UMAP: Uniform manifold approximation and projection
k-NN: k-Nearest Neighbors
WGCNA: Weighted gene co-expression network analysis
hdWGCNA: High dimensional weighted gene co-expression network analysis
MsigDB: Molecular signature database
DEGs: Differentially expressed genes
GFP: Green fluorescent protein
qPCR: Quantitative polymerase chain reaction
t-SNE: t-distributed stochastic neighbour embedding
UMI: Unique molecular identifier
TCGA: The Cancer Genome Atlas
GMP: Granulocyte-monocyte progenitor
CNA: Copy number aberration
Mps: Macrophages
CCK-8: Cell Counting Kit-8
GSEA: Gene Set Enrichment Analysis
GO: Gene Ontology
