# Supplementary material for: Single-cell transcriptome profiling of m6A regulator-mediated methylation modification patterns in elderly acute myeloid leukemia patients
Source: Mol Biomed. 2024 Dec 6;5:66. doi: 10.1186/s43556-024-00234-7 (PMC11624184; doi:10.1186/s43556-024-00234-7)
Supplement: Supplementary file 1 — Supplementary Material 1. [file 43556_2024_234_MOESM1_ESM.docx]

**Supplementary Materials for**

**Type:** Original article

**Single-cell transcriptome profiling of m^6^A regulator-mediated methylation modification patterns in elderly acute myeloid leukemia patients**

**Authors:** Zhe Wang^1#^, Xin Du^2#^, Peidong Zhang^3#^, Meiling Zhao^2^, Tianbo Zhang^2^, Jiang Liu^2^, Xiaolan Wang^2^, Doudou Chang^2^, Xiaxia Liu^4^, Sicheng Bian^5^, Xialin Zhang^2*^ and Ruijuan Zhang^2*^

1. Department of Gynecology, First Hospital of Shanxi Medical University, Taiyuan, Shanxi, 030001, China
2. Department of Hematology, Third Hospital of Shanxi Medical University, Shanxi Bethune Hospital, Shanxi Academy of Medical Sciences, Tongji Shanxi Hospital, Taiyuan, 030032, China
3. State Key Laboratory of Biotherapy and Cancer Center, West China Hospital, Sichuan University, Chengdu, 610000, China
4. Department of Hematology, Linfen Central Hospital, Linfen, 041000, China
5. Department of Medicine, Case Western Reserve University, Cleveland, OH 44106, USA

**^#^ These authors contributed equally:** Zhe Wang, Xin Du, and Peidong Zhang.

* **Addresses for corresponding author**:

Ruijuan Zhang, PhD.

Third Hospital of Shanxi Medical University, Shanxi Bethune Hospital, Shanxi Academy of Medical Sciences, Tongji Shanxi Hospital, Taiyuan, 030032, China

Email: 13593169668@163.com

Telephone number: 0086-13593169668

Xialin Zhang, PhD.

Shanxi Bethune Hospital, Shanxi Academy of Medical Sciences, Tongji Shanxi Hospital, Third Hospital of Shanxi Medical University, Taiyuan, 030032, China

Email: [charlotte007@163.com](mailto:charlotte007@163.com)

Telephone number: 0086-13903418997

**The file includes:**

Materials and methods

Figure S1 to S7

Supplementary Table 1 to Table 3

**Materials and methods**

**Construction of metacell**

The MetaCell method was employed to partition the scRNA-seq dataset into disjointed and homogeneous cell groups, referred to as metacells, using the k-NN graph algorithm. This process was carried out independently for hematopoietic stem cells (HSCs), myeloid cells, erythrocytes, and T-cells. Initially, mitochondrial genes (annotated with the prefix 'MT-') were excluded from the analysis, as these typically indicate cellular stress or death rather than reflecting cellular identity. From the count matrices of the remaining genes, feature genes with a scaled variance (variance/mean on downsampled matrices) greater than 0.08 were selected. These feature genes were used to compute cell-to-cell similarity via Pearson correlations. Based on the computed cell-to-cell similarity matrices, two balanced k-NN similarity graphs were constructed, with the parameter *K* limiting the number of neighbors for each cell. Following this, a resampling procedure was implemented, involving the resampling of 75% of the cells during each of 500 iterations. Cocluster graphs were then constructed, ensuring that the minimal cluster size was 50 cells. Ultimately, the graphs representing the metacells (along with the cells they encompass) were projected onto a two-dimensional (2D) space to facilitate the exploration of similarities between cells and metacells.

**Differential gene expression analysis**

The difference in N6-methyladenosine (m^6^A) regulator expression between cells was analysed using the FindMarkers function (Wilcoxon rank-sum test) in the Seurat package (version 4.2.0). Significant expression was based on |Log2FC| > 0.25 at a statistical significance of p< 0.05.

**Single-cell consensus weighted gene coexpression network analysis (WGCNA)**

A coexpression network analysis approach utilizing high-dimensional weighted gene coexpression network analysis (hdWGCNA) was employed to analyze the single-cell data. Initially, a bootstrapped aggregation process was applied to single-nucleus transcriptomes to construct metacells. Subsequently, metacell computations were performed, retaining new metacells for cells of the same cell type within the same sample for hdWGCNA analysis. Modules were defined based on gene expression patterns within the metacells. The first principal component of each module, termed the module eigengene, was correlated with diagnostic status and other relevant variables. Hub genes were identified using intra-modular connectivity parameters.Gene set enrichment analysis (GSEA) was conducted using the EnrichR platform to further characterize the functional significance of the identified modules and hub genes.

**Gene set enrichment analysis**

For pathway analysis, we selected 50 hallmark pathways from the Molecular Signatures Database (MsigDB). Initially, we generated a raw unique molecular identifier (UMI) count matrix from the Seurat object. Subsequently, the AUCell software package (version 3.16) was utilized to analyze gene set activity within the single-cell RNA-seq data.

**Cell-cell communication analysis**

A communication network through ligand-receptor interactions was constructed using CellChat software (version 1.1.3). Initially, ligand-receptor pairs expressed in fewer than 10% of cells were excluded. The average expression of each ligand-receptor pair across different cell types was then compared, with statistical significance set at *p* < 0.05.

**NicheNet**

Ligand-target links between interacting cells were predicted by integrating their expression data with prior knowledge of signaling and gene regulatory networks using the NicheNet package (https://github.com/saeyslab/nichenetr).

Fig. S1


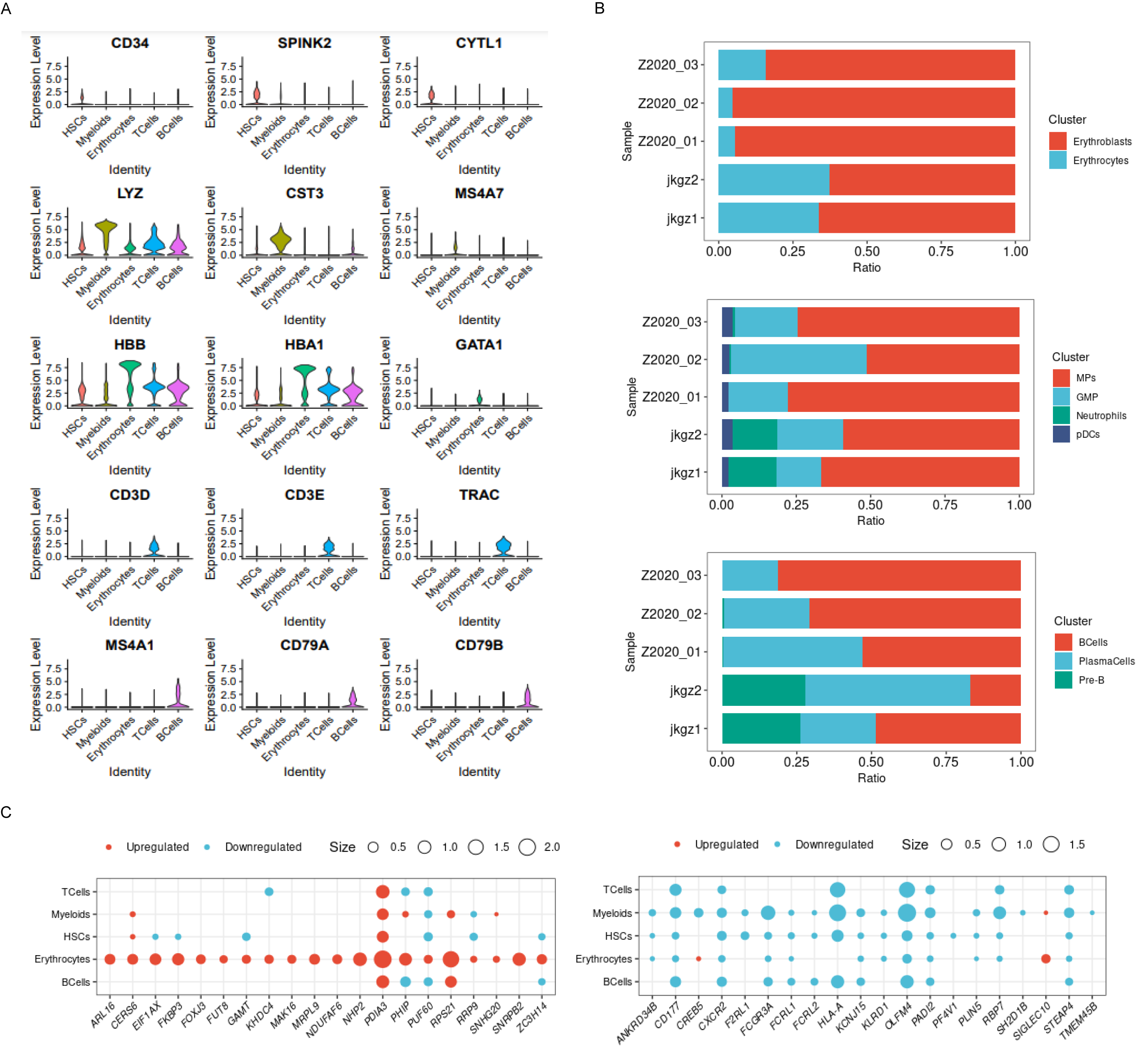


**Fig. S1 Aberrant Gene Expression Profiles in cell type-specific manners in AML.** **A** Violin plots displaying the expression of canonical markers for each cell type (control, n = 2 patients; AML, n = 3 patients). The horizontal line within each box represents the median, and the top and bottom of each box indicate the 75th and 25th percentile. Two-sided Wilcoxon rank-sum test was applied to test the significance of the gene expression with p-value < 0.05. HSCs, Myeloids, Erythrocytes, TCells, BCells. **B** The fraction of cells in normal samples and patient samples for Erythrocytes, Myeloids and BCells. **C** Dot plots showing the relative expression change of specific genes across different cell types. The size indicates the Log2FC values (AML/control).Red, up-regulation; blue, down-regulation.

Fig. S2

**Fig. S2 Distribution and expression profiles of m^6^A across individual cell types in AML of 12 cell types. A** Respective cell-type assignments in Uniform Manifold Approximation and Projection (UMAP) resulting from the batch-corrected principal component analysis (PCA) matrix using Harmony, showing 12 cell types of the AML. **B and C** Data of the 12 malignant cell subclusters of 54,809 cells from 5 samples (from left to right): the fraction of cells originating from each patient (B), the number of cells(C). **D** Analysis of m^6^A score for 12 cell types. **E** UMAP plot of m^6^A regulators expression in normal and AML samples. **F** **and** **G** Heatmap for differences in the expression of m^6^A regulators in diverse cell types between normal and AML samples (F). Dot plots showing the expression of m^6^A regulators in diverse cell types in AML (G). Red, up-regulation; blue, down-regulation. **H** Proportion of m^6^A regulators expression in 12 diverse cell types.

Fig. S3

**Fig. S3 Association of AML pathways with m^6^A regulators in diverse cell types.** **A** Heatmap for differences in the expression of m^6^A regulators in diverse cell types between normal and AML. Red, up-regulation; blue, down-regulation. **B-D** The three enrichment from the GSVA results, and associated with m^6^A regulators (B) negative senescence.(C)AML activation, and (D)positive myeloid differentiation (p < 0.05) t-value > 0 indicates that the pathway was activated (red), t-value < 0 indicates that the pathway was inhibited(blue); **E** **and** **G** The correlation analysis between the key pathways or genes and m^6^A regulators expression in GMP. **F** The correlation between the key pathways and m^6^A regulators in HSCs. **H** **and I** The genes of telomerase holoenzyme complex assembly had significant correlation with *WTAP* expression level; The genes of monocyte differentiation had significant correlation with *FTO* expression level. the blue column indicated negative and the red column indicate positive correlation. **J and K** Spearman correlation between *FTO* and monocyte differentiation. Spearman correlation between *WTAP* and monocyte differentiation. And Enrichment analysis for PINK module by GO enrichment.

Fig. S4

**Fig. S4** ***FTO* upregulated in TCells caused negative regulation of T cell-mediated immunity. A** Reclustering of TCells reveals different distribution of malignant. **B** The correlation analysis between the key pathways and m^6^A regulators expression in TCells. **C** Spearman correlation between *FTO* and TCells mediated immune response to tumor cell. **D** The genes of positive regulation of myeloid leukocyte cytokine production involved in immune response had significant correlation with *FTO* expression level. **E** Spearman correlation between *FTO* and replicative senescence. **F** The correlation between the key genes and m^6^A regulators in TCells. **G** Networks of WGCNA module which included *FTO* in TCells; **H** Enrichment analysis for *FTO* module by GO enrichment. **I** Communication and ligand-receptor interaction between TCells and neighboring cells. **J** NicheNet was used to analyze the expression of ligands and receptors to identify the intercellular communication patterns between TCells and HSCs.

Fig. S5

**Fig. S5 m^6^A regulators regulated cell-type specific functions in diverse cell types. A** NicheNet was used to analyze the expression of ligands and receptors to identify the intercellular communication patterns between Myeloids and Erythrocytes. **B** NicheNet was used to analyze the expression of ligands and receptors to identify the intercellular communication patterns between Myeloids and HSCs. **C** NicheNet was used to analyze the expression of ligands and receptors to identify the intercellular communication patterns between Myeloids and TCells. **D** NicheNet was used to analyze the expression of ligands and receptors to identify the intercellular communication patterns between HSCs and Myeloids. **E** NicheNet was used to analyze the expression of ligands and receptors to identify the intercellular communication patterns between TCells and Myeloids.

Fig. S6

**Fig. S6 External data verification through 17 AML patients by scRNA-Seq.**

(A) TSNE plot of 35203 cells from 17 AML patient samples. (B) Heatmap shows the m^6^A regulator expression in diverse cell types of AML. Red, high expression; Blue, down expression. (C) The proportion of cells expressing the twelve key m^6^A regulators in different cell types. (D-E) The TSNE shows distinct *YTHDF2* and *IGF2BP2* expression levels in diverse cell types in AML. (F) The violin plot shows that m^6^Ascore is ubiquitously expressed in diverse cell types. (G-J) Scatterplot showed the expression of m^6^A regulators in relation to the expression of key gene or signatures. (K) Networks of WGCNA module which included *YTHDF2* (Up). Barplot of *YTHDF2* module enriched function pathway. The X-axis is -log(p-value). The Y-axis is the different pathways in which genes are involved (Down).

Fig. S7

**Fig. S7 *FTO* regulated THP-1 and MV411 cells invasion and oxidative stress.**

(A) Cell invasion abilities of THP-1 and MV411 cells were determined by transwell assays after transfection in 48 hours. (B) The invasion in THP-1 and MV411 cells transfected with sh-NC and sh-FTO expression vector, ** and *** represent *p* <0.01 and *p* <0.001, respectively (THP-1 cell on the left and MV411 cell on the right of the bar graphs). (C) The ROS level was analyzed by flow cytometer after being transfected with indicated plasmids.

**Supplementary Table 1.**

The clinical characters of 3 elderly AML patients and 2 healthy individuals

| Patient | Age(y) | Sex | Tissue | Blast(%) | Stage at  analysis | Karyotype | Gene |
| --- | --- | --- | --- | --- | --- | --- | --- |
| 1 | 67 | M | BM | 33 | New  diagnosis | 46, XY, del(20)(q4) [3]/46, XY[7] | FLT3 -ITDlow/NPM1 |
| 2 | 62 | M | BM | 50 | New  diagnosis | 46, XY[20] | CEBPAdm/GATA2 |
| 3 | 61 | F | BM | 22.5 | New  diagnosis | 45, X, -X[1] | IDH2 /NPM1 |
| 4 | 51 | M | BM | - | Normal | 46, XY[20] | - |
| 5 | 43 | M | BM | - | Normal | 46, XY[20] | - |

**Supplementary Table 2.**

shRNA sequence

| Name | shRNA sequence(5’ to 3’) |
| --- | --- |
| shFTO-1 | ACCTGAACACCAGGCTCTTTA |
| shFTO-2 | CAACAGGAACCTTGGATTATA |

**Supplementary Table 3.**

General information for 17 participants of independent database from GSE235923.

| **Number** | **Group** | **Age(years)** | **PB Blast(%)** | **BMA Blast(%)** |
| --- | --- | --- | --- | --- |
| 1 | AML | 12 | 90.5 | 96.4 |
| 2 | AML | 13 | 25.0 | 68.6 |
| 3 | AML | 6.3 | 21.0 | 85.8 |
| 4 | AML | 1.5 | 0.0 | 59.0 |
| 5 | AML | 17.8 | 83.0 | 91.1 |
| 6 | AML | 10.8 | 8.0 | 91.2 |
| 7 | AML | 1.6 | 85.6 | 81.1 |
| 8 | AML | 12.3 | 84.1 | 89.8 |
| 9 | AML | 1.6 | 37.0 | 66.2 |
| 10 | AML | 12.1 | 95.7 | 96.4 |
| 11 | AML | 7.7 | 27.0 | 29.0 |
| 12 | AML | 17.5 | 32.5 | 29.5 |
| 13 | AML | 17.3 | 53.0 | 65.8 |
| 14 | AML | 16.7 | 10.0 | 40.3 |
| 15 | AML | 1.5 | 3.0 | 92.8 |
| 16 | AML | 16.1 | 5.0 | 88.8 |
| 17 | AML | 14.8 | 45.7 | 90.8 |

AML: acute myeloid leukemia, PB: Peripheral blood, BMA: Bone marrow aspirate.
